# Two-stage screening for obstructive sleep apnea in the primary practice setting

**DOI:** 10.1007/s11325-024-03142-w

**Published:** 2024-09-10

**Authors:** Andrej Pangerc, Marija Petek Šter, Leja Dolenc Grošelj

**Affiliations:** 1 Department of Family Medicine, Faculty of Medicine, University of Ljubljana, 1000 Ljubljana, Slovenia; 2 Institute of Clinical Neurophysiology, Division of Neurology, University Medical Centre Ljubljana, 1000 Ljubljana, Slovenia; 3 Department of Neurology, Faculty of Medicine, University of Ljubljana, 1000 Ljubljana, Slovenia

**Keywords:** Family medicine, Obstructive sleep apnea, Primary practice setting, STOP-BANG questionnaire, Home sleep apnoea testing (HSAT), Two-stage screening

## Abstract

**Purpose:**

To evaluate the effectiveness of a two-stage screening model for obstructive sleep apnea (OSA) in primary care that combines the STOP-BANG questionnaire (SBQ) with an automated home sleep apnea test (HSAT).

**Methods:**

This cross-sectional study was conducted from August 2018 to August 2022 in four Slovenian primary care practices. It included 153 randomly selected patients aged 18 to 70 years who visited the practice for any reason. Participants completed the SBQ and underwent HSAT with type III polygraphy on the same night. The HSAT recordings were scored automatically and by an experienced, accredited somnologist.

**Results:**

There was a strong correlation between manual and automated HSAT scorings for the detection of OSA (Pearson’s r = 0.93). Cohen’s kappa was 0.80 for OSA (respiratory event index (REI) ≥ 5) and 0.77 for OSA severity categorization. The two-stage model demonstrated sensitivity of 64%, a specificity of 97.4%, a positive predictive value (PPV) of 96.0%, a negative predictive value (NPV) of 73.8% and an accuracy of 81.1% for any OSA (REI ≥ 5). For moderate to severe OSA (REI ≥ 15), the model showed 72.7% sensitivity, 96.7% specificity, 85.7% PPV, 92.8% NPV and 91.5% accuracy.

**Conclusions:**

The two-stage model for OSA screening combining the SBQ and automated HSAT was shown to be effective in primary care, especially for moderate and severe OSA. This method provides a practical and efficient approach for the early detection of OSA.

## Introduction

Obstructive sleep apnea (OSA) is a major cause of excessive daytime sleepiness, contributing to a reduced quality of life and is associated with an increased incidence of hypertension, type 2 diabetes mellitus, atrial fibrillation, heart failure, coronary heart disease, motor vehicle accidents, stroke, and death [1].

The diagnosis of OSA requires formal testing, i.e. a sleep study [2]. In-lab polysomnography (PSG) is the gold standard but is time-consuming and inconvenient. Home sleep apnea testing (HSAT) offers a more convenient home-based alternative [3] and is cheaper then PSG that is why HSAT is being increasingly utilized in clinical practice [4]. PSG is recommended over HSAT for patients with certain medical conditions, including cardiorespiratory disease, neuromuscular-related respiratory muscle weakness, chronic opioid use, and stroke history. HSAT is not well-validated for these conditions and may miss or underestimate sleep disorders [4].

HSAT is typically carried out using a type III modified portable polygraph (PG) which measures air flow, respiratory effort, oxygen saturation and heart rate breathing [3].

Sleep study results are expressed as the apnea–hypopnea index (AHI) for PSG and the respiratory event index (REI) for HSAT, which indicate the average number of apneas and hypopneas per hour of sleep or bedrest respectively [5]. Based on the number of these events, OSA is categorized as mild (5 ≤ AHI/REI < 15), moderate (15 ≤ AHI/REI < 30) and severe (AHI/REI ≥ 30) [6]. HSAT’s disadvantages include the lack of an EEG, making it unable to distinguish between wakefulness and sleep, potentially underestimating OSA severity by about 10%. Additionally, HSAT is only suitable for diagnosing OSA, not other sleep disorders [7]. Nevertheless, Meta-analyses show that type 3 PG devices are both sensitive and specific for diagnosing OSA in uncomplicated patients with a high pretest probability of the condition [7].

Treatment is generally recommended for patients with moderate and severe OSA, as well as for persons mild OSA with symptoms of OSA or coexisting conditions such as hypertension, ischemic heart disease, or a history of stroke [8].

The scoring of sleep studies is traditionally performed manually by a somnologist or a qualified technician. Automated approaches have been gaining. However, automated scoring is not yet commonplace [9].

Various questionnaires, including the widely used STOP-BANG questionnaire (SBQ), assess the likelihood of obstructive sleep apnea (OSA). An SBQ score of ≥ 3 indicates a high risk of OSA. [4].

A meta-analysis by Chiu et al. found that the STOP-BANG questionnaire (SBQ) is the most sensitive and has the best diagnostic odds ratio for detecting mild, moderate, and severe OSA. It is also quick and easy to complete [10]. We previously published details on the translation, adaptation, test–retest reliability, internal consistency and validation of the Slovenian SBQ in a sleep laboratory [11] and in primary practice [12].

In the current paradigm of care, the primary care provider is best placed to identify patients with OSA symptoms [13].

OSA has the characteristics of a disease that lends itself to screening, i.e. it is common, has a long latency period, is identifiable and has an effective treatment [14]. Opinions on OSA screening are divided. Chang and Goldberg et al. noted that while STOP-BANG is sensitive, there’s no evidence of long-term health effects of screening versus no screening. The harms of screening seem limited, but the cost-utility trade-off remains unassessed [7]. The U.S. Preventive Services Task Force advises against routine OSA screening due to a lack of high-quality research and insufficient evidence of health benefits [1]. On the other hand, the American Acadimy of Sleep Medicine (AASM) [3] and the American Heart Association recommend screening in certain groups of comorbid patients [15]. PSG is the gold standard for diagnosing OSA, but it is unsuitable for screening due to its high cost, complexity, and inaccessibility [16].

HSAT (type 3 PG) is not suitable for screening due to its time-consuming nature. Questionnaires, on the other hand are not sufficient for diagnosis due to their low accuracy rate and a formal sleep study is still required [3].

The aim of our study was to evaluate the automated scoring of HSAT and to test a two-stage model for OSA in primary care practise based on the SBQ questionnaire followed by HSAT with automated scoring.

## Methods

### Study design and setting

In our cross-sectional study conducted in four Slovenian family practices, patients were randomly selected from the first ten daily visitors using a random number protocol.

Physicians educated patients on obstructive sleep apnea, emphasizing its impact on driving safety. After the discussion, detailed written information was provided. Patients could ask questions and were assured they could withdraw from the study at any time without explanation. Written consent was acquired.

### Participants

We recruited patients aged 18–70 years who visited participating physicians for any reason. Exclusion criteria included sleep-disordered breathing, regular use of sedatives, tranquilizers, or opioids (including tramadol), heart failure, neuromuscular disease, psychiatric disorders, severe COPD (stage D), use of psychoactive substances and excessive alcohol consumption.

### Data collection

Patient inclusion took place between August 1, 2018, and August 1, 2022.

A primary practice nurse coordinated communication and scheduling. Upon arrival, patients completed a short questionnaire along with the Slovenian SBQ. The nurse then provided detailed instructions on how to use the HSAT device.

All participants, regardless of their SBQ, performed HSAT at home later that day.

### HSAT equipment and scoring

HSAT was performed with Alice NightOne (Phillips Respironics Murrysville, Pennsylvania, USA), a type 3 PG with seven data channels, including effort belt, cannula, pulse oximeter and body position sensor with microphone. OSA was categorised as mild (5 ≤ REI < 15), moderate (15 ≤ REI < 30) or severe (REI ≥ 30). All recordings were manually scored by a European accredited somnologist (using Sleepware G3 V 3.9.1) following the American Academy of Sleep Medicine standards [17]. Manual scoring was considered the reference standard for assessing automated scoring and the two-stage model.

The two-phase model was evaluated as follows:

- The screening was considered negative if the SBQ score was less than 3.
- If the SBQ score was 3 or higher, the screening was considered positive if the automated REI was 5 or higher, and negative if the REI was less than 5.

Regardless of the SBQ score, all recordings were automatically (using Sleepware G3 V 3.9.1) and manually scored to allow a comparison between automated scoring and manual scoring. The lights-out and lights on periods for both scorings were identical. This period was determined using a post-test questionnaire, completed in the morning following the HSAT, in corelation with body position as recorded by HSAT device. Recordings of less than 3 h were considered inadequate.

### Final dataset

A total of 159 patients were included. Fifteen had unsuitable PG recordings due to issues like nasal cannula misplacement, pulse oximeter malfunction, or short recordings. Nine recordings were repeated, while six patients refused repeat recording. The final analysis included 153 patients, with a mean age of 49.5 years (± 13.0 years), 73 (47.7%) were male.

### Statistical analysis

Patient characteristics were summarized using means for numeric variables and frequencies (%) for categorical variables. The non-normally distributed manual and automated REI were compared using the Wilcoxon signed-rank test and Pearson’s r. We used scatter plots, rain diagrams, and Bland–Altman plots to visualize the comparison between automated and manual HSAT scores. Sensitivity, specificity, PPV, and NPV were calculated. Statistical analyses were performed with SPSS version 15.0 and JASP version 0.16.4.

### Large language models

The authors utilized ChatGPT-4o (OpenAI, San Francisco, California) to refine and condense certain portions of the text. Following the use of this tool, the authors thoroughly reviewed and edited the content as necessary and assume full responsibility for the final content of the publication.

## Results

### Description of the population

153 participants were included, of whom 75 (49.0%) were diagnosed with OSA (REI ≥ 5). Detailed are provided in Table 1.

**Table 1:** Descriptive statistics for the primary practice setting sample according to manual HSAT scoring

	All	Normal (REI < 5)	Mild OSA (5 ≤ REI < 15)	Moderate OSA (15 ≤ REI < 30)	Severe OSA (REI ≥ 30)
n	153	78 (51%)	42 (27.5%)	23 (15%)	10 (6.5%)
Age (years)	49.7 ± 13.1	43.6 (± 12.9)	55 (± 11.7)	58.5 (± 7)	54.1 (± 7.6)
Sex (m)	73 (47.7%)	30 (38.5%)	21 (50%)	13 (56.5%)	9 (90%)
BMI (kg/m^2^)	28.0 ± 4.9	26.1 (± 4.1)	29.1 (± 4.9)	30.6 (± 4.4)	33 (± 5.4)

Legend: *BMI* body mass index, *kg* kilogram, *OSA* obstructive sleep apnea, *REI* respiratory event index, *m* male, *m*^*2*^ square metre, *n* number of participants

### Correlation between automated and manual HSAT scoring

A total of 75 patient had a REI ≥ 5 (49.0%), whereas the automatic scoring has overestimated the number of sleep apnea patients with (REI ≥ 5) up to 88 (56,3%).

**Fig. 1:**
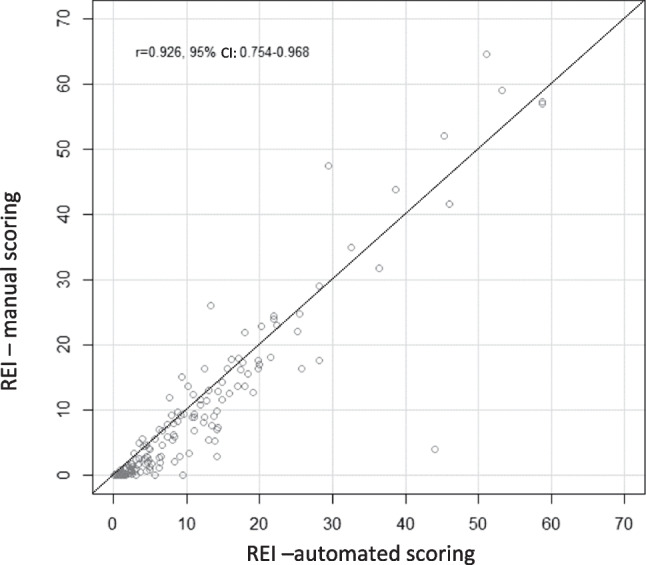
Scatter plot of REI based on automated and manual HSAT scoring. Legend: CI = confidence interval, r = Pearson's r, REI = respiratory event index

**Fig. 2:**
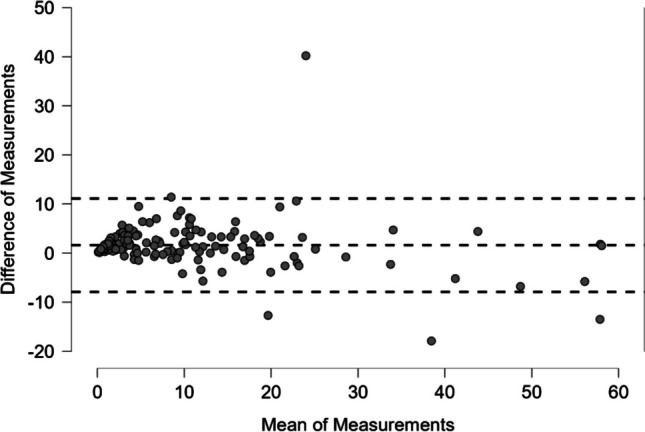
Bland–Altman plot comparing REI from automated and manual scoring of HSAT

REI showed a strong correlation with Pearson’s r = 0.926 (95% CI: 0.745–0.968, p < 0.001) between manual and automated scoring (see Fig. 1 for scatter plot). Nevertheless, the automated REI was statistically significantly higher with a median difference of 1.55 (95% CI: 1.15–1.95, p < 0.001) based on the Hodges-Lehmann estimate and 1.607 (limits of agreement of -7.90 to 11.11) based on Bland–Altman analysis (see Fig. 2 for Bland–Altman plot).

We found strong agreement between automated and manual HSAT scoring for diagnosing OSA (REI ≥ 5), with a Cohen’s kappa of 0.80 (95% CI: 0.71 – 0.90), indicating excellent interrater reliability. Of the 15 discrepancies, automated scoring incorrectly identified OSA in 14 cases. One mild OSA case (manual REI of 5.5 and automated REI of 4.0) was missed by automated scoring. One recording had an automated REI of 41.1 and a manual REI of 3.2 due to a missing thoracic belt signal but was not excluded as manual scoring was still possible.

Diagnostic characteristics of automated scoring of HSAT for any OSA is given in Table 2.

**Table 2:** Diagnostic characteristics of automated scoring of HSAT for any OSA (REI ≥ 5)

		95% credible interval	
	Estimate	Lower	Upper
Sensitivity	0.965	0.916	0.992
Specificity	0.818	0.729	0.890
PPV	0.822	0.736	0.893
NPV	0.964	0.914	0.992
Accuracy	0.886	0.834	0.929

Legend: *HSAT* home sleep apnea testing, *NPV* negative predictive value = $\frac{true\;negative}{true\;negative + false\;negative}$, *OSA* obstructive sleep apnea, *PPV* positive predictive value = $\frac{true\;positive}{true\;positive + false\;positive}$, *REI* respiratory event index, *sensitivity* $\frac{true\;positive}{true\;positive + false\;negative}$, *specificity* $\frac{true\;negative}{true\;negative + false\;positive}$

The interrater agreement for categorization into normal, mild, moderate, and severe OSA was substantial, with a Cohen’s Kappa of 0.77 (95% CI: 0.69 – 0.86).

Diagnostic characteristics of automated scoring of HSAT for moderate and severe OSA is given in Table 3.

**Table 3:** Diagnostic characteristics of automated scoring of HSAT for moderate and severe OSA (REI ≥ 15)

		95% credible interval	
	Estimate	Lower	Upper
Sensitivity	0.884	0.772	0.960
Specificity	0.946	0.900	0.978
PPV	0.813	0.675	0.918
NPV	0.969	0.935	0.990
Accuracy	0.933	0.892	0.965

Legend: *HSAT* home sleep apnea testing, *NPV* negative predictive value = $\frac{true\;negative}{true\;negative + false\;negative}$, *OSA* obstructive sleep apnea, *PPV* positive predictive value = $\frac{true\;positive}{true\;positive + false\;positive}$, *REI* respiratory event index, *sensitivity* $\frac{true\;positive}{true\;positive + false\;negative}$, *specificity* $\frac{true\;negative}{true\;negative + false\;positive}$

### Two-stage model

Of 59 patients with a positive SBQ, 50 had concordant positive automated HSAT results, with 49 confirmed OSA cases (manual REI ≥ 5.0). Eight had a positive SBQ with negative automated HSAT and no OSA on manual scoring. One had a positive SBQ, a negative automated HSAT, yet mild OSA (manual REI 5.5).

The contingency table for the two-stage screening model for any OSA (SBQ ≥ 3, REI ≥ 5) in the family medicine clinic, where the thresholds were SBQ ≥ 3 and automated REI ≥ 5, can be found in Table 4 and the diagnostic characteristics in Table 5.

**Table 4:** Contingency table for the two-stage screening model for any OSA (REI ≥ 5) in the family medicine clinic

Manual REI ≥ 5				
		Positive	Negative	∑
Two-stage screening (SBQ ≥ 3 and auto REI ≥ 5)	Positive	48	2	50
	Negative	27	76	103
	∑	75	78	153

Legend: *OSA* obstructive sleep apnea, *REI* respiratory event index

**Table 5:** Diagnostic characteristics of the two-stage screening model for any OSA (REI ≥ 5)

		95% credible interval	
	Estimate	Lower	Upper
Sensitivity	0.640	0.521	0.748
Specificity	0.974	0.910	0.997
PPV	0.960	0.858	0.989
NPV	0.738	0.675	0.792
Accuracy	0.811	0.739	0.869

Legend: *accuracy* $\frac{true\;positive + true\;negative }{true\;positive + true\;negative + false\;positive + false\;negative}$, *HSAT* home sleep apnea testing, *NPV* negative predictive value = $\frac{true\;negative}{true\;negative + false\;negative}$, *OSA* obstructive sleep apnea, *PPV* positive predictive value = $\frac{true\;positive}{true\;positive + false\;positive}$, *REI* respiratory event index, s*ensitivity* $\frac{true\;positive}{true\;positive + false\;negative}$, *specificity* $\frac{true\;negative}{true\;negative + false\;positive}$

The interrater agreement between the two-stage model (SBQ ≥ 3, REI ≥ 5) and manual scoring for any OSA (REI ≥ 5) was substantial, with a Cohen’s Kappa of 0.62 (95% CI: 0.50 – 0.77).

The diagnostic characteristics for the two-stage model for moderate and severe OSA (REI ≥ 15) are given in Table 6.

**Table 6:** Diagnostic characteristics of the two-stage model, with a threshold of SBQ ≥ 3 and auto REI ≥ 15, for moderate and severe OSA (REI ≥ 15)

		95% credible interval	
	Estimate	Lower	Upper
Sensitivity	0.727	0.545	0.867
Specificity	0.967	0.917	0.991
PPV	0.857	0.691	0.942
NPV	0.928	0.881	0.958
Accuracy	0.915	0.859	0.954

Legend: *accuracy* $\frac{true\;positive + true\;negative }{true\;positive + true\;negative + false\;positive + false\;negative}$, *HSAT* home sleep apnea testing, *NPV* negative predictive value = $\frac{true\;negative}{true\;negative + false\;negative}$, *OSA* obstructive sleep apnea, *PPV* positive predictive value = $\frac{true\;positive}{true\;positive + false\;positive}$, *REI* respiratory event index, *sensitivity* $\frac{true\;positive}{true\;positive + false\;negative}$, *specificity* $\frac{true\;negative}{true\;negative + false\;positive}$

The interrater agreement between the two-stage model, with a threshold of SBQ ≥ 3 and auto REI ≥ 15, and manual scoring of HSAT for moderate and severe OSA (REI ≥ 15) was substantial, with a Cohen’s Kappa of 0.73 (95% CI: 0.60 – 0.87).

Diagnostic characteristics I for the two-stage model for severe OSA (REI ≥ 30) are given in Table 7.

**Table 7:** Diagnostic characteristics of the two-stage model, with a threshold of SBQ ≥ 3 and auto REI ≥ 30, for severe OSA (REI ≥ 30)

		95% credible interval	
	Estimate	Lower	Upper
Sensitivity	0.900	0.556	0.998
Specificity	0.867	0.800	0.918
PPV	0.321	0.229	0.430
NPV	0.992	0.951	0.999
Accuracy	0.869	0.805	0.918

Legend: *accuracy* $\frac{true\;positive + true\;negative }{true\;positive + true\;negative + false\;positive + false\;negative}$, *HSAT* home sleep apnea testing, *NPV* negative predictive value = $\frac{true\;negative}{true\;negative + false\;negative}$, *OSA* obstructive sleep apnea, *PPV* positive predictive value = $\frac{true\;positive}{true\;positive + false\;positive}$, *REI* respiratory event index, *sensitivity* $\frac{true\;positive}{true\;positive + false\;negative}$, *specificity* $\frac{true\;negative}{true\;negative + false\;positive}$

The interrater agreement between the two-stage model, with a threshold of SBQ ≥ 3 and auto REI ≥ 30, and manual scoring of HSAT for severe OSA (REI ≥ 30) was fair, with a Cohen’s Kappa of 0.42 (95% CI: 0.22 – 0.61).

## Discussions

Our study found excellent corelation between Philips Respironics Sleepwere G3 version 3.9.1 automated scoring and manual scoring of HSAT recorded with by Alice NightOne, a type III portable polygraphy device. The two-stage model for OSA screening, in which a positive SBQ is followed by automated scoring, has good diagnostic properties, and agrees with manual scoring of HSAT.

In our study, automated HSAT scoring yielded slightly higher REI (1.6/hour) than manual scoring but showed excellent correlation (Pearson’s r = 0.93). Kristiansen et al. found similar results (1.1/hour higher, Pearson’s r = 0.96), Cachada et al. (Pearson’s r = 0.91) [18], and Labarca (Pearson’s r = 0.95). However, Cohen’s kappa showed moderate agreement (0.58) for OSA diagnosis and weak agreement (0.33) for severity [19]. In our study, interrater reliability was excellent for OSA presence (Cohen’s kappa = 0.80) and substantial for severity categorization (Cohen’s kappa = 0.77).

The slightly higher REI from automated scoring could misclassify some borderline cases as mild OSA. However, this is of limited clinical relevance, as asymptomatic mild OSA patients are usually not started on CPAP therapy unless they have other conditions [8], furthermore CPAP shows no cardiovascular benefits [20] and has a high treatment failure rate in this group [21]. Importantly, no significant cases were missed.

Automated HSAT scoring had a sensitivity of 0.97 and specificity of 0.82 for any OSA (REI ≥ 5), slightly higher than Kristiansen’s findings for the Nox T3 (sensitivity 0.93, specificity 0.71) [22]. Our statistics were affected by an outlier classified as severe OSA by automated scoring but normal by manual scoring due to a missing thoracic belt signal. This underscores the need for high-quality signals in all channels for reliable automated scoring in clinical practice.

The excellent characteristics of automated HSAT in our study, along with strong correlations in other studies, underscores the potential of automated scoring. Our findings and Peñacoba’s views suggest that automated HSAT scoring could be a viable solution in primary care, where time constraints and limited scoring experience exist [23].

Provided an adequate file sharing platform and the willingness of sleep laboratories and clinics to cooperate type 3 PG recordings made in primary practice for screening could be used for definite diagnosis of OSA. Over time, automated scoring of HSAT may enable primary care physicians to make diagnoses independently.

To the best of our knowledge this is the first time a two-stage screening for OSA in the family practice setting, using type 3 PG, was performed. Our two-stage model showed good agreement with manual scoring (Cohen’s kappa of 0.62) for any OSA (REI ≥ 5). It had a sensitivity of 0.64, specificity of 0.97, and accuracy of 0.81, outperforming the Slovenian SBQ questionnaire alone (sensitivity 0.65, specificity 0.87). For moderate to severe OSA (REI ≥ 15), crucial for identifying those who benefit most from treatment, it achieved a kappa of 0.73, sensitivity of 0.73, specificity of 0.97, and accuracy of 0.92.

Chai-Coetzar’s two-stage screening using a questionnaire and type 4 polygraphy (pulse oximetry with desaturation detection) showed 88% sensitivity and 82% specificity for severe OSA (AHI ≥ 30) [24]. Gurubhagavatula et al. tested various two-stage models in patients with arterial hypertension, finding them effective for severe OSA but less so for milder forms [25]. Unlike type 3 PG that we used, type 4 PG, used in these studies, does not monitor airflow and chest movements, potentially underestimating REI as it cannot detect apneas without desaturation or arousals and is therefore less accurate [26].

If we had referred patients based on our two-stage model instead of the SBQ alone, referrals would have decreased by 15.3%. Whilst not directly comparable Peñacoba found that type 4 PG reduced referrals by 55.1% compared to a four-question screening questionnaire [27]. Our model missed one OSA patient (1.7%) compared to the SBQ alone. This patient was positive on the SBQ, negative with automated scoring, and had an REI of 5.5 on manual scoring of HSAT.

Whilst opinions on whether to screen, whom to screen and how to screen remain divided, the authors agree with Miller [28] who believes that family medicine clinics are the ideal place for OSA screening, where it could take place as part of regular screening of high-risk patients.

Our study had several limitations. First, we restricted participant age to 70 years to enhance engagement and comprehension of instructions, potentially affecting the sample’s representativeness. Compliance with CPAP therapy decreases in patients over 65[29] so these patients would potentially benefit less in practice.

The COVID-19 pandemic extended our recruitment period from one to four years, including a two-year hiatus, slowing recruitment upon resumption. Limited funding for patient recruitment also posed a challenge.

Using type 3 PG with manual scoring instead of type 1 PSG was another limitation. However, this aligned with standard care and available resources, as type 3 PG is common in clinical practice. We excluded patients taking sedatives, opioids, or tranquilizers, and those with heart failure, neuromuscular disease, or COPD stage D, as these conditions are better evaluated with type 1 PSG according to AASM guidelines.

Using type 3 PG with manual scoring instead of type 1 PSG was a limitation. One reason is that HSAT cannot distinguish between central and obstructive sleep apnea. However, this approach aligns with standard care and available resources, as type 3 PG is common in clinical practice. Given that central sleep apnea is present in only 0.4% of the general population [30] and we excluded patients taking sedatives, opioids, or tranquilizers, as well as those with heart failure, neuromuscular disease, or COPD stage D, where central apnea is more common, we can be confident in the validity of the method used.

## Conclusions

The waiting times for sleep studies are becoming increasingly long, and many patients remain undiagnosed. It is evident that the current approach to diagnosing and treating OSA must change. We need a method to identify patients with OSA in the primary care setting without generating numerous false positives or missing significant cases, particularly those with moderate and severe OSA, for whom therapy has proven benefits. Our two-phase screening has demonstrated its potential to achieve this. The excellent correlation between manual and automated scoring of type 3 PG in primary care is a crucial prerequisite for the potential use of such devices in a busy primary care setting, as primary care physicians cannot be expected to score PG manually. Furthermore, by using type 3 PG, which is an accepted and widespread method of establishing a diagnosis of OSA, we could someday send recordings rather than patients to, often far off, sleep laboratories for confirmation of diagnosis keeping in mind the inherent limitations of HSAT.

## Data Availability

Data will be made available on reasonable request. I confirm I have included a data availability statement in my main manuscript file.

## Declarations

### LLM statement

During the preparation of this manuscript, the authors utilized Chat GPT-4 (OpenAI, San Francisco, California, USA) for language enhancement and to condense specific sections of the paper. After using this tool, the authors reviewed and edited the content as needed and take full responsibility for the content of the publication.
